# Carbon content-tuned martensite transformation in low-alloy TRIP steels

**DOI:** 10.1038/s41598-019-44105-6

**Published:** 2019-05-17

**Authors:** Y. F. Shen, X. X. Dong, X. T. Song, N. Jia

**Affiliations:** Key Laboratory for Anisotropy and Texture of Materials (Ministry of Education), School of Material Science and Engineering, Northeastern University, Shenyang, 110819 China

**Keywords:** Mechanical properties, Metals and alloys

## Abstract

Ultrahigh strength and good ductility are obtained for two low-alloy transformation-induced-plasticity steels fabricated by the quenching and partitioning (Q&P) processing, respectively. Compared to 0.19 wt.% C steel in which γ → α′-martensite transformation is the dominant mechanism under deformation, the relatively high C content of austenite in 0.47 wt.% C steel is responsible for the transformation from γ to ε-martensite, suggesting that the transformation is not solely determined by the stacking fault energy. The study shows that during the Q&P process, strong and ductile steels with specific transformation procedures can be obtained by adjusting volume fraction and carbon content of the retained austenite.

## Introduction

Transformation-induced-plasticity (TRIP) steels are indispensable materials for the application in automotive body construction, owing to their high strength and superior ductility. Medium- and high- Mn steels are the most promising candidates with the TRIP effect and thus show excellent mechanical properties. The strengthening mechanism for those materials is based on the transformation of the metastable austenite induced by deformation, i.e., the face-centered cubic (FCC) structured γ-phase characterized by a high work hardening capacity to achieve very high strength together with exceptional ductility^1^ to martensite characterized by the hexagonal close-packed (HCP) ε-phase and/or body-centered cubic (BCC) α′-phase^2–7^.

Till now, research indicates that there exist two mechanisms of martensitic transformation, i.e., the stress-assisted and the strain-induced transformations^8^. For FCC materials, deformation mechanisms and mechanical properties are intrinsically related to the stacking faults energy (SFE), as this value determines whether twinning, martensite transformation or dislocation slip dominates deformation. In general, the SFE below 20 mJ/m^2^ promotes a direct transformation from austenite to ε-martensite and then to α′-martensite, while the SFE larger than 20 mJ/m^2^ leads to activation of twinning and transformation to α′-martensite^8,9^. Chemical composition strongly affects the stability of metastable RA at ambient temperature^10^. The high austenite stability can be achieved by increasing the concentration of C and Mn, as the both elements act as strong austenite stabilizers^11^. During intercritical annealing the size effect of austenitic grains and the partitioning of Mn into austenite mainly promotes the stability of austenite^12,13^. Then austenite stability can be maintained through partitioning the austenite stabilizers (such as Mn and C) between ferrite and austenite^14,15^.

Inspired by these studies, the present work aims to clarify the relationship between heat-treatment processing, microstructures, transformation procedures and mechanical properties of two low-alloy TRIP steels. The chemical compositions of the steels are 0.47C-0.45Si-1.76Mn-1.52Al and 0.19C-0.30Si-1.76Mn-1.52Al, respectively. We find that by adjusting volume fraction and carbon content of the retained austenite during the quenching and partitioning (Q&P) processing^2,16,17^, strong and ductile steels owing to their specific transformation procedures can be obtained.

## Experimental Procedure

### Materials

TRIP steels with the nominal chemical compositions of 0.47C-0.45Si-1.76Mn-1.52Al (wt. %, named as medium-carbon steel) and 0.19C-0.30Si-1.76Mn-1.52Al (wt.%, named as low-carbon steel) were fabricated, respectively. The latter steel has been previously studied in ref.^18^. and here used as a comparison with the steel with a C content of 0.47 wt.%. The steels were melted and homogenized at 1200 °C, then rolled at 950 °C with five passes to reach a total thickness reduction of 80%, and further cold rolled to 70% thickness reduction by six passes. Subsequently, medium-carbon steel was heat-treated by using a two-step Q&P processing, i.e., intercritical annealing at 820 °C for 300 s, quenching at 250 °C for 8 s and partitioning (bainitic holding) at 420 °C for 180 s. Low-carbon steel was treated by using one-step Q&P processing including 820 °C intercritical annealing for 300 s, quenching to 420 °C and partitioning for 180 s. Under a heating rate of 150 °C/s the steels were heated to reach the intercritical region (820 °C), and held for 120 s. Finally, medium-carbon and low-carbon steels were quenched to 250 °C and 420 °C, respectively. A cooling rate of −40 °C/s was used for the steels.

### Mechanical testing and microstructural characterization

Uniaxial tension along the rolling direction (RD) of the as-fabricated steels was performed at room temperature. The strain rate was 5 × 10^−3^ s^−1^. During loading strains were measured with a contactless laser extensometer. Samples used for the X-ray diffraction (XRD) analysis were etched with a solution of nitric acid (30 vol.%) and hydrochloric acid (70 vol.%). The XRD measurements were performed on an X’Pert diffractometer equipped with the Co K_α1_ anode. The carbon concentration of RA was determined with an assumption that the lattice parameters are affected by the chemical compositions^19^, using the method described in supplemental materials. Electron backscattering diffraction (EBSD) measurements were made for the rolled planes of the steels, using a SU-70 Hitachi field-emission scanning electron microscope (SEM) at an operating voltage of 20 kV. EBSD specimens were electro-polished with an electrolyte consisting of perchloric acid (10 vol.%), ethanol (80 vol.%) and deionized water (10 vol.%) for 20 s at 20 V. The scanning step was 0.1 µm and the EBSD data were analyzed using the OIM software. Deformation microstructures were further analyzed with a field-emission-gun Tecnai transmission electron microscope (TEM) operated at 200 kV. TEM specimens cut from the homogeneously deformed regions were prepared by mechanically polishing down to 50 µm thickness and punching into 3 mm diameter discs. Subsequently, twin-jet electropolishing at −10 °C was conducted using an electrolyte with perchloric acid (10 vol.%) and ethanol (90 vol.%) at 32 V.

## Results and Discussion

**Figure 1:**
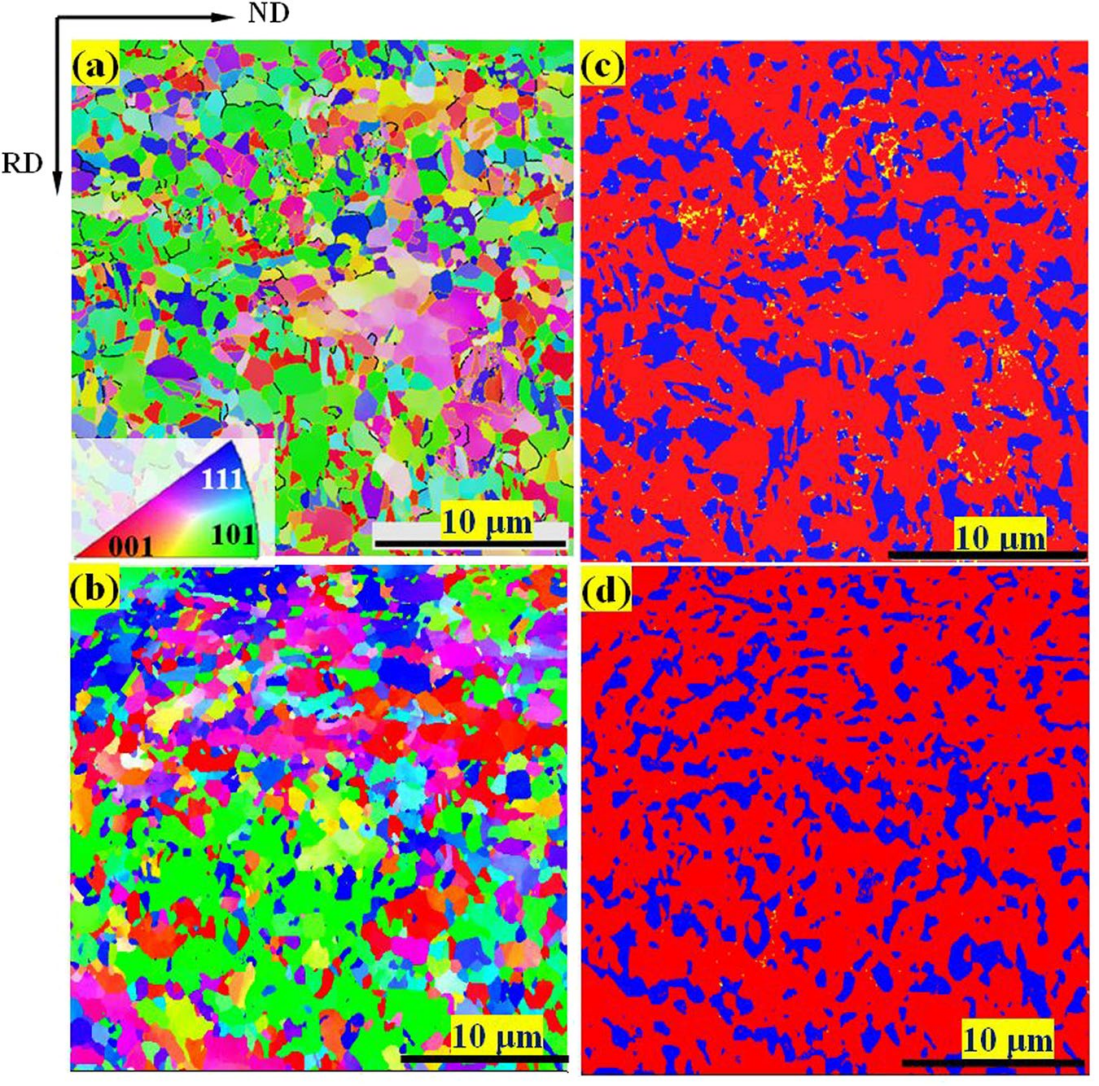
Inverse-pole-figure (IPF) maps illustrating the morphologies on planes composed of normal direction (ND) and rolling direction (RD) for the as-fabricated 0.47 wt.% C steel (**a**) and the 0.19 wt.% C steel (**b**), respectively. The IPF maps show the grain orientations with respect to RD. The phase distribution maps corresponding to the IPF maps (**a**,**b**) are shown in (**c**,**d**), respectively, with blue for FCC phase (retained austenite), red for BCC phase (ferrite and α′-martensite) and yellow for HCP phase (ɛ-martensite).

Grain morphologies and phase distributions of the as-fabricated steels were characterized by EBSD (Fig. 1). For medium-carbon steel the average grain size is ~3 μm, which is slightly smaller than that in 0.19 wt.% C steel (~5 μm). The RA volume fraction is as high as 32 ± 2% in medium-carbon steel, which is significantly higher than that in low-carbon steel (~21 ± 2%). The XRD analysis show that the carbon concentration of RA in 0.47 wt.% C steel is 1.63 wt.% whilst that in 0.19 wt.% C steel is 1.17 wt.%. The intercritical temperature governs the concentration of alloying elements. Carbon element is the stabilizer of austenite and C atoms will diffuse from ambient ferrite to austenite during annealing. This leads to the high C concentration in austenite. Nevertheless, the initial carbon contents were different between the two steels. The larger C content facilitated the diffusion from ferrite to austenite, leading to the higher concentration of carbon in medium-carbon steel. On the other hand, medium-carbon steel undergoes the two-step Q&P process including the extra quenching at 250 °C for 8 s. Thus, the reverse phase transformation may be another responsible mechanism. Consistent observation has been reported in a 1.04 Cu-bearing steel undergone the three-step heat treatment, in which copper was enriched inside the austenite and a large copper content (15~19 wt.%) was found as the reverse transformation occurred^20^.

**Figure 2:**
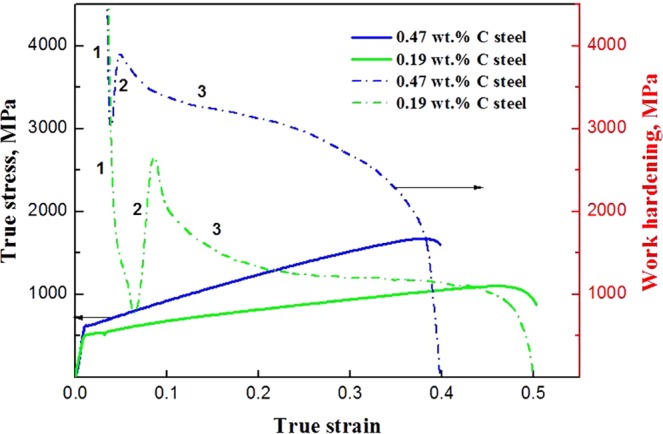
True stress – strain curves and the corresponding work-hardening rate – true strain curves for the steels.

True stress-strain curves of the steels are presented in Fig. 2, in which a combination of high ultimate tensile strength and superior elongation can be seen for the both steels. Medium-carbon steel shows that the yield strength (*σ*_y_) is 630 MPa, the ultimate tensile strength (*σ*_UTS_) is 1680 MPa, and the elongation to failure is 0.38, respectively. Low-carbon steel shows the decreased *σ*_y_ (520 MPa) and *σ*_UTS_ (1100 MPa), but the increased maximum elongation of 0.50. The work-hardening curves in Fig. 2 show that work-hardening ability of the medium-carbon steel is significantly higher than that of the low-carbon steel.

**Figure 3:**
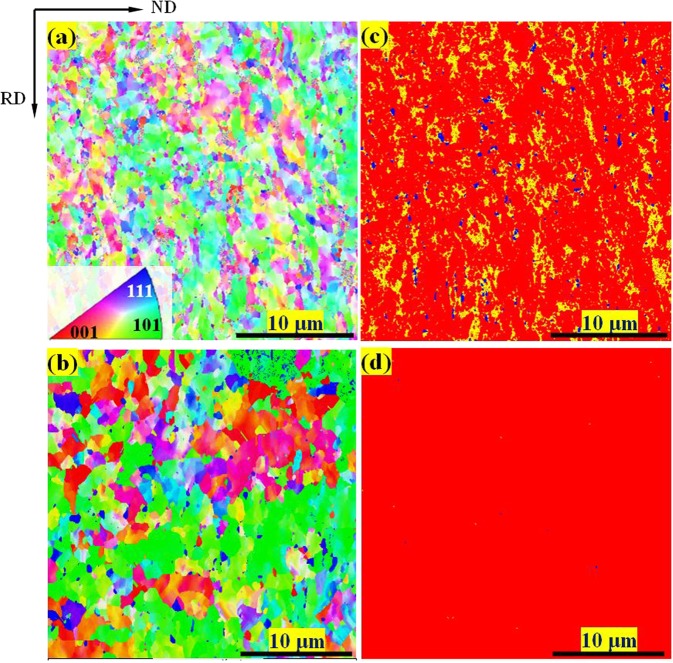
Inverse-pole-figure (IPF) maps illustrating the deformed morphologies on planes composed of ND and RD for the 0.47 wt.% C steel (**a**) and the 0.19 wt.% C steel (**b**), respectively. The IPF maps show the grain orientations with respect to RD (// tensile axis). The phase distribution maps corresponding to the IPF maps (**a**,**b**) are shown in (**c**,**d**), respectively, with blue for FCC phase (austenite), red for BCC phase (ferrite and α′-martensite) and yellow for HCP phase (ɛ-martensite).

Microstructures of the steels after tensile tests characterized by EBSD are shown in Fig. 3. No obvious variation in grain size appears in either steel compared to that prior to loading. However, significant differences can be identified between the phase distribution maps. For medium-carbon steel the BCC phases composed of ferrite and α′-martensite are dominant. Meanwhile, the HCP phase tangled with a few FCC phase is uniformly distributed around the BCC phases. The ɛ-martensite and the RA show the volume fractions of 30% and 3%, respectively (Fig. 3c and Table 1). During tensile deformation, the transformed volume fraction of RA to ɛ-martensite is as high as 27% whilst that transformed to α′-martensite is only 2%. In contrast, in the deformed low-carbon steel the total volume fraction of BCC phases is as high as ~98%, without the occurrence of intermediate ε-martensite (Fig. 3d and Table 1). This suggests that the majority of RA has been transformed to α′-martensite during tensile loading.

**Table 1:** Volume fractions of austenite, ɛ-martensite and α′-martensite in the steels at different strains.

0.47 wt.% C	True strain	0	0.1	0.2	0.3	0.4	0.5
	γ	32	/	16	/	3	/
	ɛ	3	/	15	/	30	/
0.19 wt.% C	γ	21	18	14	7	2	0
	α′	0	5	9	16	19	21

The statistics for austenite and ɛ-martensite are obtained from EBSD whilst that for α′-martensite are obtained from TEM.

**Figure 4:**
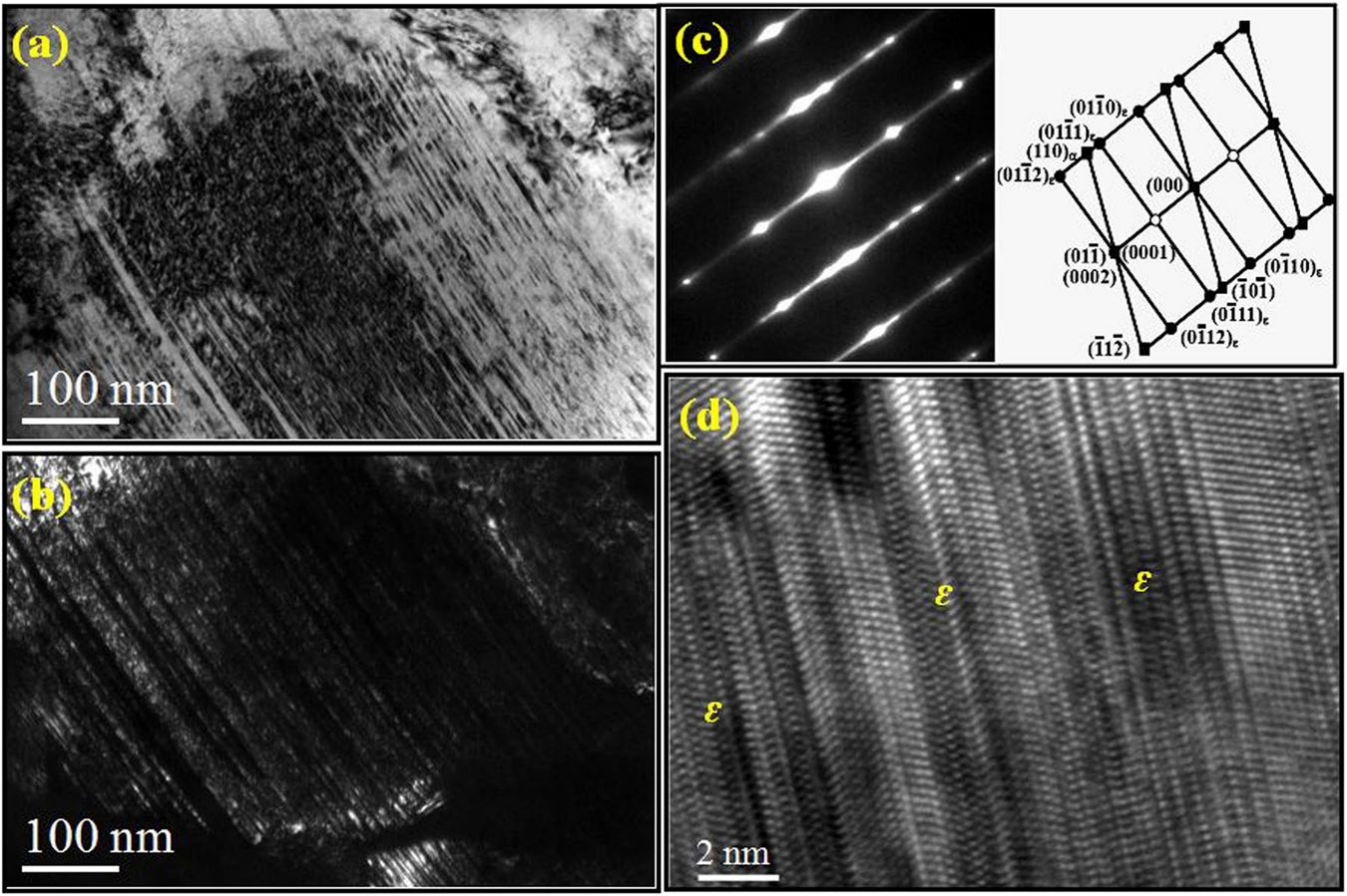
Bright-field (**a**) and dark-field (**b**) TEM images showing the γ-austenite → ɛ-martensite transformation in the 0.47 wt.% C steel under a true strain of 0.38. (**c**) The corresponding SAED pattern and indexed pattern of the orientation relationship between ɛ and α. (**d**) HRTEM image showing the morphology of ɛ-martensite.

**Figure 5:**
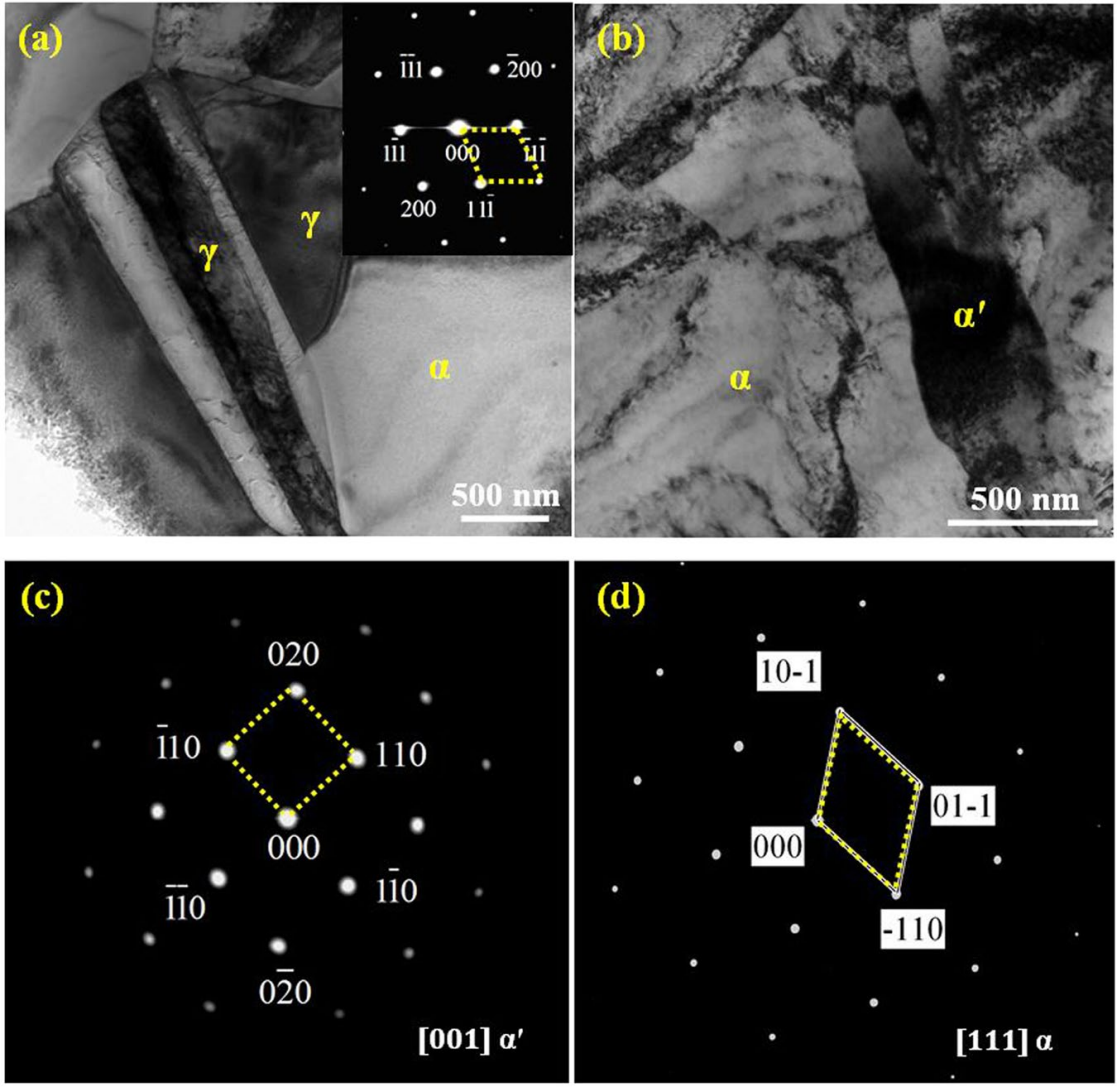
TEM images of the 0.19 wt.% C steel before (**a**) and after deformation (**b**) under a true strain of 0.50. The inset on the right top of (**a**,**c**,**d**) show the corresponding SAED patterns and indexed patterns of the retained austenite (RA), α′-martensite and ferrite, respectively.

TEM observations show the ɛ-martensite dominated microstructures in medium-carbon steel after deformation (Fig. 4). The high density of dislocations pile up between the intervals of ε-plates (Fig. 4a,b), suggesting that ε-martensite act as obstacles to slip. This is interesting because previous investigation on austenitic steels showed that during the transformation from austenite to α′-martensite, ε-martensite merely exists as an intermediate phase^21^. Here, streaks in the selected-area-electron-diffraction (SAED) patterns are related to the formation of HCP phase (Fig. 4c). The ε-martensite and ferrite coexist following a relationship of ${(0001)}_{\varepsilon }//{(110)}_{\alpha }$ with the zone axis of ${[2\bar{1}\bar{1}0]}_{\varepsilon }//{[111]}_{\alpha }$, namely the Kurdjumov-Sachs (K-S) misorientation relationship is fulfilled^22^. When the slip of stacking faults (SFs) is superposed on every second {111}γ, HCP structure can be formed. Therefore, it is conceivable that the irregular overlapping SFs gradually evolve into a regular sequence. High-resolution TEM (HRTEM) image for the region in Fig. 4a shows that the deformed microstructure consists of a bundle of thin ε-martensite plates. In contrast, low-carbon steel exhibits the different deformation behavior. Comparing the microstructures before and after tensile testing (Fig. 5a,b), one can see that RA (γ) has been transformed to α′-martensite under deformation. High density of dislocations appears in both ferrite and α′-martensite, contributing to the high ultimate tensile strength and good ductility of the bulk material. The SAED patterns of the γ phase (Fig. 5a) suggest an FCC lattice. Figure 5c,d reveal the SAED patterns of the α′-martensite and ferrite, taken along a zonal axis of [001]_α′_ and [111]α, respectively.

The stress-strain curves indicate that the *σ*_UTS_ increases with increasing austenite from the steel with 0.19 wt.% C to that with 0.47 wt.% C. However, the total elongation of the steels goes along an inverse way. According to ref.^23^, martensitic transformation may lead to an increased work-hardening rate, delaying necking and thus effectively improves the ductility. As was demonstrated by our previous work^11^, the RA with the larger carbon concentration showed the elevated stability, enabling the slower but more gradual martensitic transformation during straining. Here the RA in medium-carbon steel is too stable to transform to α′-martensite because of its high carbon content. Both of the situations lead to the small increase in elongation^8^. Here, it is reasonable that as the carbon content in RA is moderate, low-carbon steel shows the larger elongation. This is related to the gradual phase transformation during the whole straining up to a true strain of 0.5, leading to the continuous strain-hardening behavior. It has been well established that uniform plastic deformation represents the resistance to necking for a material, i.e., a measure of the strain-hardening capability^24^. SEM images and elemental distribution maps detected by electron probe microanalysis (EPMA) of the steels can be found in the supplemental materials (Fig. S1). In the both steels RA is enriched in C, Al and Mn, illustrating that those elements are the stabilizers of austenite. However, the level of carbon content in medium-carbon steel is higher than that in low-carbon steel, despite of the identical levels of Al and Mn concentrations. Here we prove that RA is with the identical contents of Al and Mn but the significantly different carbon concentration between the steels. Therefore, the different phase transformation procedures should be mainly determined by the carbon concentration of RA.

**Figure 6:**
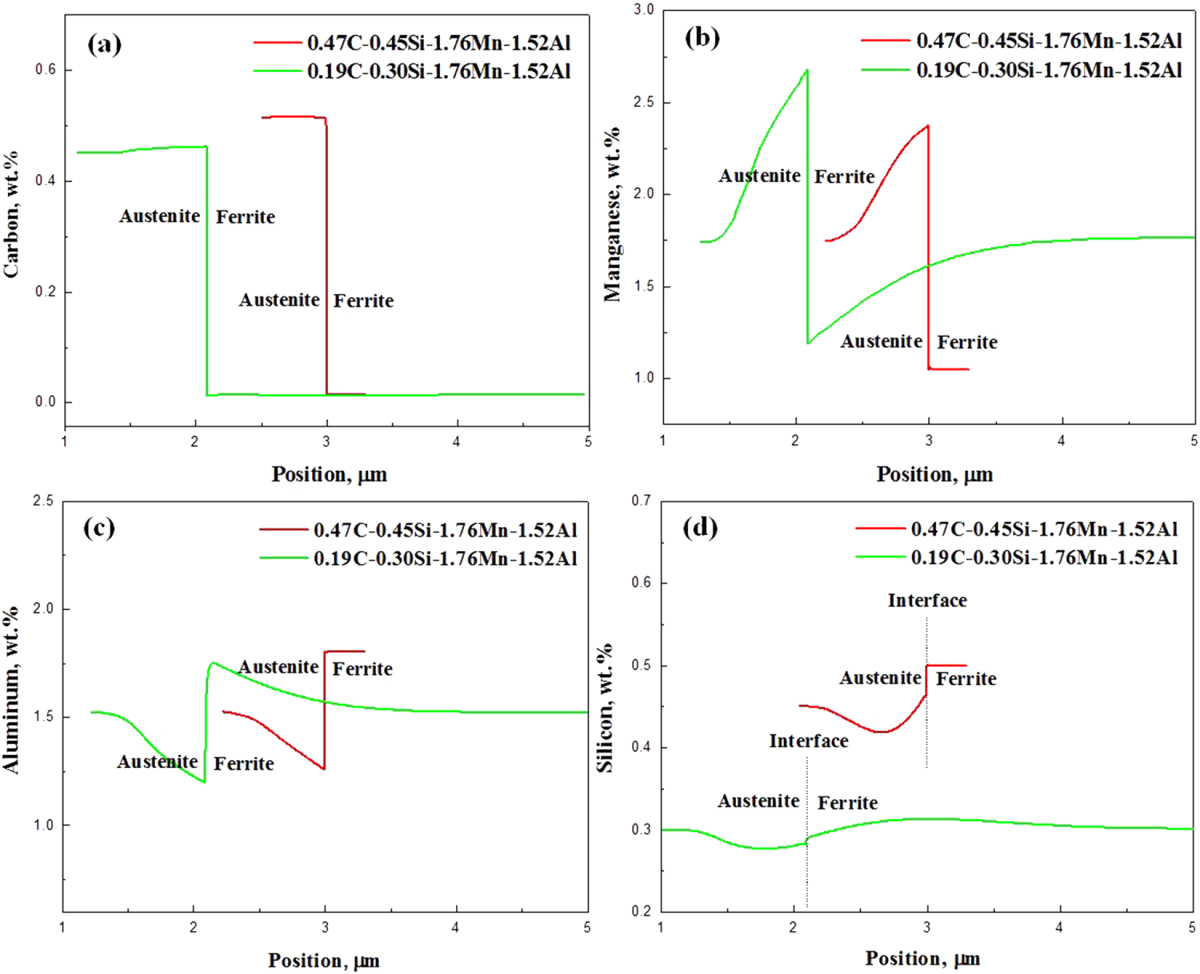
DICTRA calculated alloying element (**a**) C, (b) Mn, (**c**) Al and (**d**) Si distributions in the steels after intercritical annealing at 820 °C for 300 s. The calculation shows that the interface between RA and ferrite moves toward the ferrite side and the displacements of the interface are different between the steels, leading to a wider layer of RA in the 0.47 wt.% C steel with relatively high carbon content.

**Figure 7:**
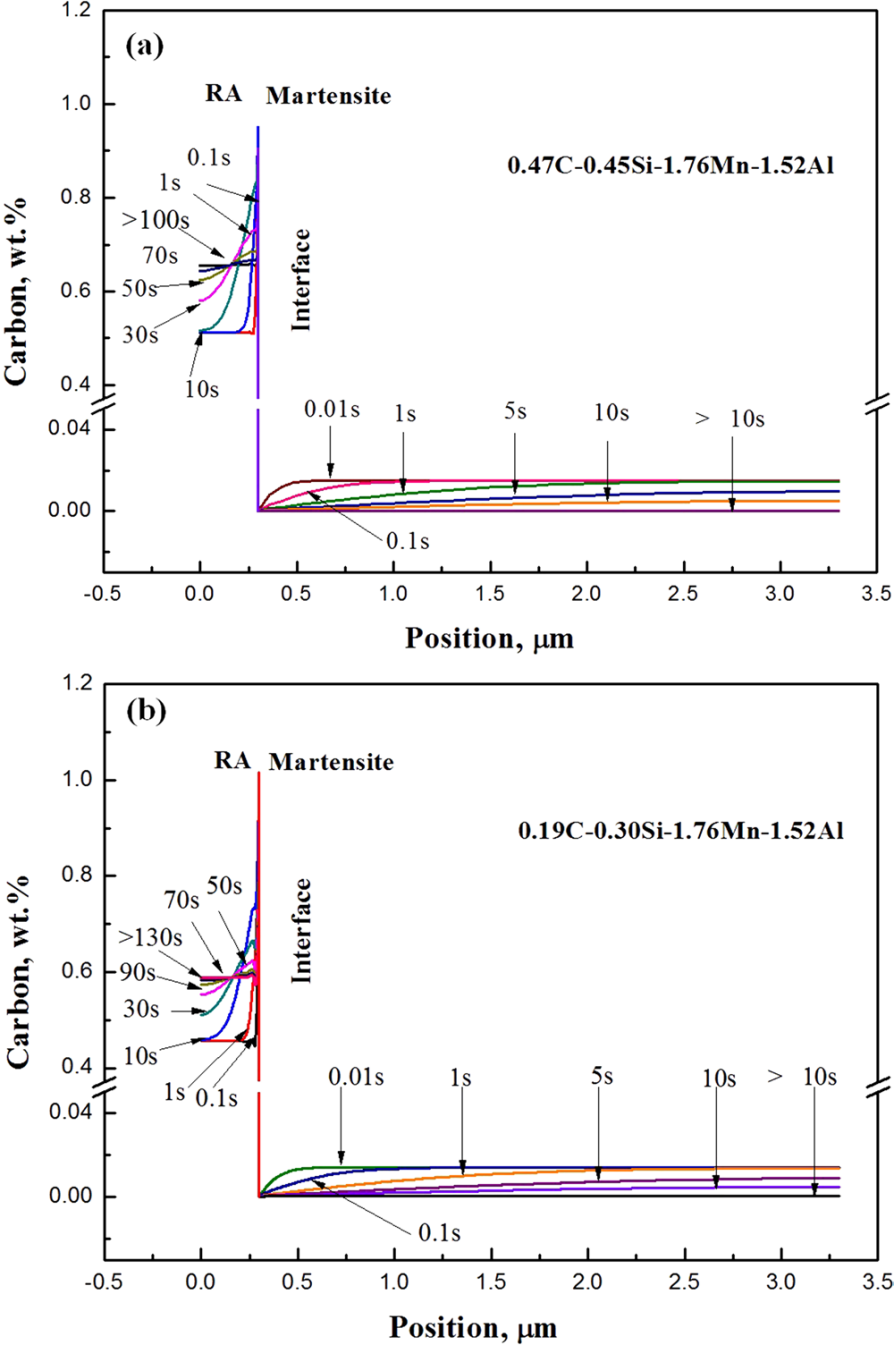
DICTRA calculated distribution and evolution of carbon in the steels during tempering at 420 °C for 180 s: (**a**) 0.47C-0.45Si-1.76Mn-1.52Al steel and (**b**) 0.19C-0.30Si-1.76Mn-1.52Al steel.

To reveal the partitioning of alloying elements under intercritical annealing and bainitic holding, DICTRA simulations invoking thermodynamic database TCFE6 and mobility database MOBFE1^25,26^ were performed. Details of the model establishment are given in the supplemental materials (Fig. S2). The objective of partitioning during Q&P is to obtain RA enriched with carbon. Austenite nucleates at an interface of martensite during the intercritical annealing, hence the alloy compositions of RA and martensite are the same as that at the initial stage of annealing. After the intercritical annealing of medium-carbon steel, in austenite C and Mn are enriched whilst in ferrite Al and Si exhibit the higher concentrations, as shown in Fig. 6. For low-carbon steel annealed at the identical condition, the similar trends appear for C, Mn and Al, whereas the distribution of Si varies between the steels because of its different content in the steels. The objective of bainitic holding is also to obtain the carbon-enriched RA. The calculated carbon distribution during bainitic holding is presented in Fig. 7. The calculations show that the homogenization of carbon in RA is completed at around 100 s and 130 s in medium-carbon and low-carbon steels, leading to the different carbon contents, i.e., 0.68 wt.% and 0.58 wt.% in RA, respectively. Therefore, partitioning at 420 °C for 180 s is sufficient for the enrichment of RA with carbon in the studied steels.

Lü *et al*.^27^ confirmed that no α′-martensite formed in a Fe-Mn-C alloy during cold rolling, even though the area fraction of ε-martensite increased from 2.7% to 33.1% as the strain increased from 22% to 51%. Generally, ε-martensite or twinning acts as an intermediate phase during the transformation from γ-austenite to α′-martensite^19,28,29^. The γ → ɛ → α′-martensite transformation occurs through a two-stage process as in advance of the final transformation to α′-martensite austenite transforms to ε-martensite first^12,30–32^. Here, our observations for medium-carbon steel support the result in ref.^27^. Namely, the formation of ε-plates is dominant during the whole period of phase transformation. To examine the role of SFE, we calculate it following the procedure provided in supplemental materials. The SFEs in the both steels increase with the increasing temperatures as well as the distances from the center to the edge of austenite (see supplemental materials, Fig. S3). For medium-carbon steel at room temperature, the SFE increase from 21 mJ/m^2^ to 32 mJ/m^2^ with the increasing distances from the center of austenite. For low-carbon steel, the value increases from 14 mJ/m^2^ to 23 mJ/m^2^ with the increasing distances. It is noteworthy that the deformation-induced transformation in medium-carbon steel shows the high room-temperature SFE exceeding the generally reported limit for high-Mn steels (i.e., ~20 mJ/m^2^), below which the formation of ε-martensite occurs^8,33,34^. According to Sato *et al*.^8^, in alloys with the SFE larger than 20 mJ/m^2^ deformation twins, instead of ε-martensite, are produced under deformation. However, the current results show that in 0.47 wt.% C steel the dominant deformation mechanism is the transformation from γ to ε-martensite. Therefore, the ε-martensite formation results from the high stability of RA with the large C content. Several studies on TWIP steels also reported inconsistent relationships between SFE range and the corresponding deformation mechanisms^35,36^.

To this stage, the larger *σ*_UTS_ of medium-carbon steel compared to low-carbon steel is attributed to the different deformation mechanisms of the steels. For medium-carbon steel, the γ → ε-martensite transformation significantly enhances work hardening at the expense of the maximum elongation. For low-carbon steel, however, the γ → α′-martensite transformation does not lead to an increased work hardening but contributes to a larger elongation. Phase transformation is determined by the stability of austenite. As previously reported that, the increasing austenite stability promotes the gradual transformation with straining and thus contributes to the high ductility^37,38^. Here, the ε-martensite acting as the final phase in the deformed medium-carbon steel is associated with the high C content and the high stability of austenite.

## Conclusions

In this work, two strong and ductile low-alloy TRIP steels with different carbon contents are fabricated by the Q&P processing, respectively. Under plastic deformation the retained austenite in 0.47 wt.% C steel transforms into ε-martensite, while in 0.19 wt.% C steel without the formation of ε-martensite as an intermediate phase, the majority of austenite transforms into α′-martensite directly. Compared to the low-carbon steel, γ → ε-martensite transformatioin in the medium-carbon steel results from high stability of the retained austenite with larger C content. Therefore, through the different phase transformation procedures that are dependent on volume fraction and carbon content of austenite the optimal combination of strength and ductility can be obtained.

## Supplementary information

Dataset 1
