# Supplementary material for: Carbon content-tuned martensite transformation in low-alloy TRIP steels
Source: Sci Rep. 2019 May 17;9:7559. doi: 10.1038/s41598-019-44105-6 (PMC6525154; doi:10.1038/s41598-019-44105-6)
Supplement: Supplementary file 1 — Dataset 1 [file 41598_2019_44105_MOESM1_ESM.docx]

**Supplemental materials**

**Carbon content-tuned martensite transformation in low-alloy TRIP steels**

Y.F. Shen^1^, X.X. Dong^1^, X.T. Song^1^, N. Jia^1,*^

1 Key Laboratory for Anisotropy and Texture of Materials (Ministry of Education), School of Material Science and Engineering, Northeastern University, Shenyang 110819, China

* Corresponding authors. E-mail addresses: [jian@atm.neu.edu.cn](mailto:jian@atm.neu.edu.cn) (N. Jia)

1. **Determination of carbon concentration of austenite**

To obtain the carbon concentration in austenite, first the volume fraction of austenite is determined by：

$V_{\gamma}=1.4I_{\gamma}(I_{a}+1.4I_{\gamma})$ (1)

where *I_γ_* and *I_α_* denote the peak intensities of austenite and ferrite, respectively. In the current work, the (200)γ, (220)γ and (311)γ peaks for RA and the (200)α and (220)α peaks for ferrite are measured, respectively.

The lattice parameter of austenite (*a_γ_*) is calculated as:

 (2)

where *n* is the number of reflections considered for austenite, and 2*θ_i_* is the X-ray scattering angle of the *hkl* reflections.

Consequently, the carbon content in RA is calculated as:

a_γ_ = 0.3556+ 0.00453C + 0.000095Mn + 0.00056 Al (3).

1. **SEM images and EPMA maps**


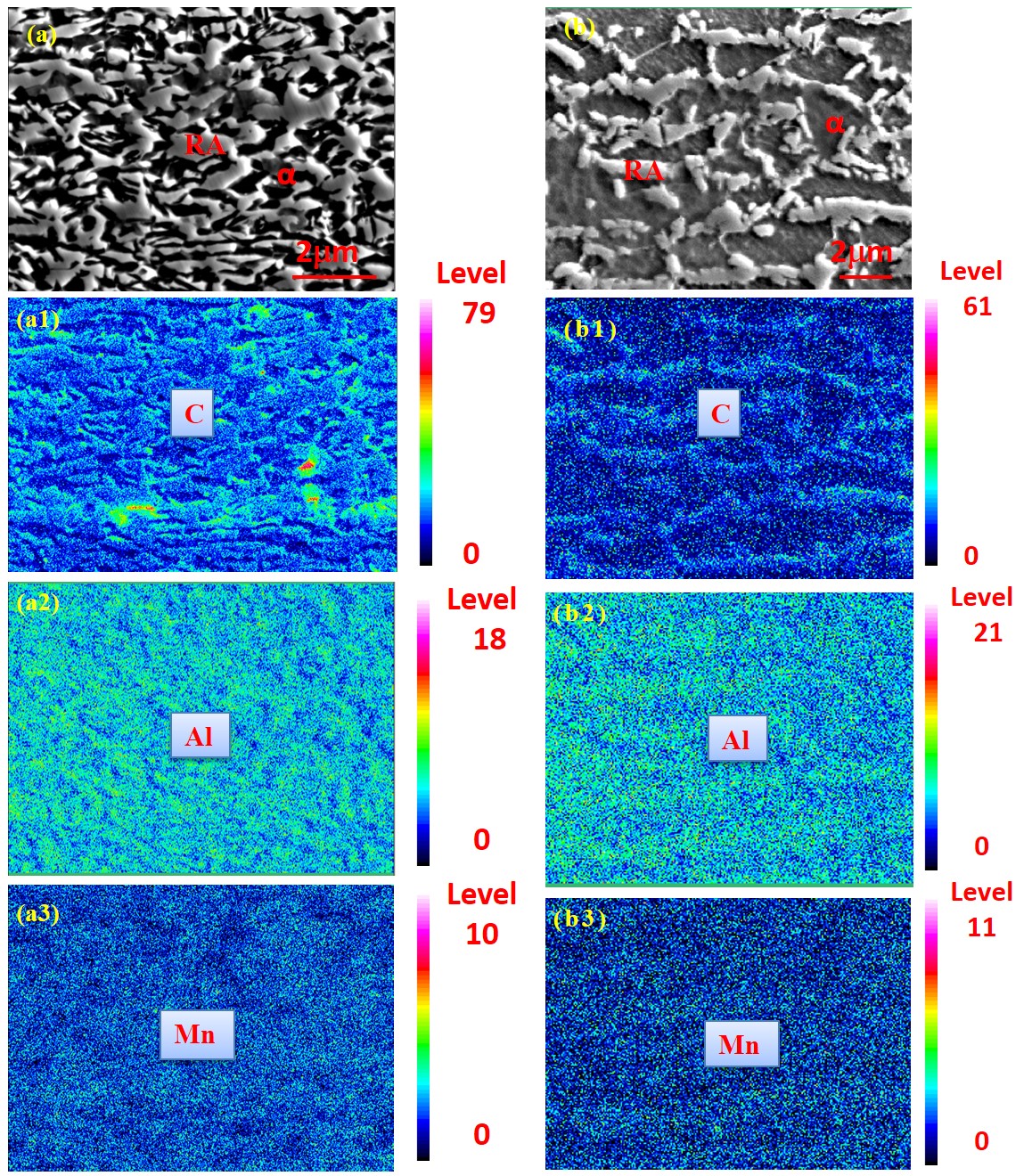


**Fig. S1** SEM images and EPMA maps over a 12 × 8 μm2 area showing the morphologies and distributions of C, Al, and Mn elements in the 0.47 wt.% C (a, a1, a2, and a3), and the 0.19 wt.% C steels (b, b1, b2, and b3), respectively. The chromaticity scale reveals the relative concentrations of individual elements.

1. **Model establishment for thermodynamic calculations**

The thermodynamic model is limited in a small space, thus the interface between austenite and martensite can be considered as planar and its moving direction is vertical to the interface [1]. The cell is divided into two regions by the interface, i.e., martensite and austenite are located on each side of the interface. Thermodynamic database as well as mobility database for the single-phase martensite cannot be obtained in the existing literatures. However, considering that the thermodynamic properties of martensite are similar to that of ferrite, martensite can be replaced by ferrite in the calculation [1]. We assume that the length of martensite and RA are infinite [2], then the model is built as one-dimensional as shown in Fig. S2. The thickness of retained austenite is considered to be 1 nm (left part in the model, there is no effect until 10 nm) and the thickness of martensite is 3 μm (right part in the model), respectively. During intercritical annealing, the phase interface moves from position 1 (before intercritical annealing) to position 2 (after intercritical annealing). Phase interface after quenching is located at position 3 of the model.

For simulating the bainitic holding process, the position of the phase interface after quenching is supposed at position 3 of the model. The displacement of the phase interface between RA and martensite can be ignored compared with that during carbon partitioning [2]. Hence the width of RA is determined as the EBSD measured average grain size of austenite. Assuming that the length of martensite and RA are infinite and ignoring the displacement of phase interface, the model is also built as one-dimensional in the quenching process.


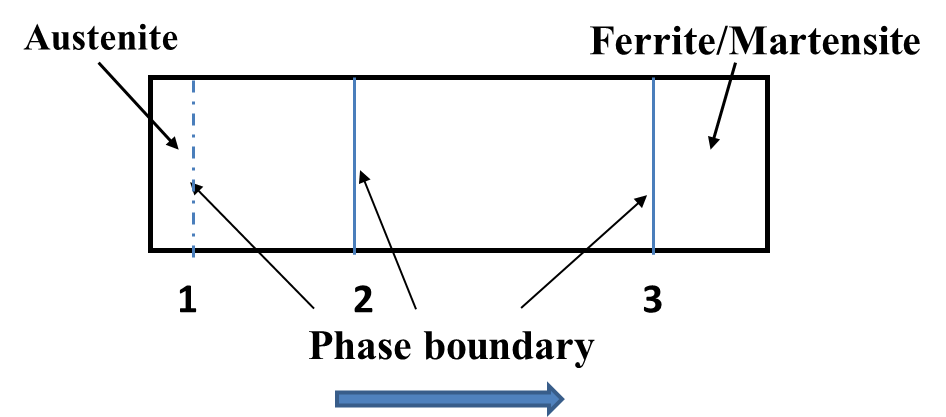


**Fig. S2** Phase boundary moving model established for DICTRA calculations.

1. **Determination of stacking fault energy (SFE)**

The SFE is calculated using the equation [3]:

γ = 2ρ${\Delta G}^{\gamma\to\varepsilon}$+2σ (4).

The molar surface density is calculated after Allain et al [4], as follows:

*ρ=*4** (5)

where *N* is Avogadro’s number and *a* is the lattice parameter of austenite, respectively. The temperature dependency of the lattice parameter is given by Andres et al [5]. The interfacial energy in the Fe-C-Si-Mn-Al system was estimated to be 10 ± 5 mJ/m^2^ [6]. ${\Delta G}^{\gamma\to M}$ is the free energy change for *γ*/*ɛ* phase transformation, calculated by regular solution model, as follows:

${\Delta G}^{\gamma\to\varepsilon}$=$X_{Fe}{\Delta G}_{Fe}^{\gamma\to\varepsilon}$+$X_{Mn}{\Delta G}_{Mn}^{\gamma\to\varepsilon}$+$X_{Al}{\Delta G}_{Al}^{\gamma\to\varepsilon}$+$X_{C}{\Delta G}_{C}^{\gamma\to\varepsilon}$+$X_{Fe}X_{Mn}{\Delta\Omega}_{FeMn}^{\gamma\to\varepsilon}$+$X_{Fe}X_{Al}{\Delta\Omega}_{FeAl}^{\gamma\to\varepsilon}$+$X_{Fe}X_{C}{\Delta\Omega}_{FeC}^{\gamma\to\varepsilon}$+$X_{Mn}X_{C}{\Delta\Omega}_{MnC}^{\gamma\to\varepsilon}$+${\Delta G}_{mag}^{\gamma\to\varepsilon}$+${\Delta G}_{ex}$ (6)

${\Delta G}_{i}^{\gamma\to\varepsilon}$and ${\Delta\Omega}_{ij}^{\gamma\to\varepsilon}$are discussed in Refs [7,8]. ${\Delta\Omega}_{ij}^{\gamma\to\varepsilon}$is the interaction energy between the components *i* and *j*, and $X_{i}$ is molar fraction of alloying elements in the system. ${\Delta G}_{\mathrm{ex}}$ is the excess free energy related to the austenite grain size, and increases with the decreasing size of austenitic grains following:

${\Delta G}_{ex}$=170.06 *exp (*$\frac{-d}{18.55}$) (7)

where *d* is the austenite grain size in μm. In general the SFE is a constant for a given material. ${\Delta G}_{mag}^{\gamma\to\varepsilon}$ is the free energy contribution due to magnetic transition, as given by equation:

${\Delta G}_{mag}^{\gamma\to\varepsilon}$= ${\Delta G}_{mag}^{\varepsilon}$－${\Delta G}_{mag}^{\gamma}$ (8)

where $G_{mag}^{\varphi}$ is calculated by equation

$G_{mag}^{\varphi}$*=* *RT ln*($1+\frac{\beta^{\theta}}{\mu B}$)*f*($\frac{T}{T_{N}^{\theta}}$)*, θ=y, ε*  (9).

Here *β^θ^* and $T_{N}^{\theta}$, and *μB* are the magnetic moment, the Néel temperature of phase *θ*, and the Bohr magneton, respectively, as calculated by Eqs. (10)–(13):

${\frac{\beta}{\mu B}}^{\gamma}$= 0.7$X_{Fe}$+0.62$X_{Mn}$－0.64$X_{Fe}X_{Mn}$－4$X_{C}$ (10)

${\frac{\beta}{\mu B}}^{\varepsilon}$=0.62$X_{Mn}$－4$X_{C}$ (11)

$T_{N}^{\gamma}$= 251.71 +6.81$X_{Mn}$－11.51$X_{Al}$－15.57$X_{Si}$－17.4$X_{C}$ (12) $T_{N}^{\varepsilon}$= 580$X_{Mn}$ (13)

$X_{i}$ is molar fraction of elements *i* in the system, $T_{N}^{\varepsilon}$is in Kelvin, and *f* is a polynomial function.

Using the fixed interface energy $\sigma^{\gamma/\varepsilon}$of 10 mJ/m^2^, the SFE of the studied steels are calculated by DICTRA based on chemical composition distribution after intercritical annealing. The size of austenitic grains are assumed as 3 μm and 5 μm for the 0.47 wt.% C and 0.19 wt.% C steels, respectively.

1. **Calculated SFE**

**Fig. S3** Distribution of SFE in austenite for the 0.47 wt.% C and the 0.19 wt.% C steels after intercritical annealing at different temperatures.

**References:**

[1] O. Dmitrieva, D. Ponge, G. Inden, et al. Chemical gradients across phase boundaries between martensite and austenite in steel studied by atom probe tomography and simulation. Acta Mater. 2011;59:364–374.

[2] S. Liu, Z. Xiong, H. Guo, et al. The significance of multi-step partitioning: Processing-structure-property relationship in governing high strength-high ductility combination in medium-manganese steels. Acta Mater. 2017;124:159–172.

[3] S. Curtze, V.-T. Kuokkala, A. Oikari, et al. Thermodynamic modeling of the stacking fault energy of austenitic steels. Acta Mater. 2011;59:1068–1076.

[4] S. Allain, J.-P. Chateau, O. Bouaziz, et al. Correlations between the calculated stacking fault energy and the plasticity mechanisms in Fe-Mn-C alloys, Mater. Sci. Eng. A. 2004;387:158–162.

[5] A. Garcia-Junceda, C. Capdevila, F.G. Caballero, et al. Dependence of martensite start temperature on fine austenite grain size. Scripta Mater. 2008;58:134–137.

[6] W. Yang, C. Wan. The influence of aluminium content to the stacking fault energy in Fe-Mn-Al-C alloy system. Mater. Sci. 1990;25:1821–1823.

[7] A. Saeed-Akbari, J. Imlau, U. Prahl, et al. Derivation and variation in composition-dependent stacking fault energy maps based on subregular solution model in high-manganese steels. Metall. Mater. Trans. A 2009;40:3076–3090.

[8] A. Dumay, J.-P. Chateau, S. Allain, et al. Influence of addition elements on the stacking-fault energy and mechanical properties of an austenitic Fe-Mn-C steel. Mater. Sci. Eng. A. 2008;483:184–187.
